# A Too Common Affront to the Profession

**Published:** 1893-12

**Authors:** 


### A TOO COMMON AFFRONT TO THE PROFESSION.

About a year since the *Jonrnal of the American Medical Association,* in an editorial article, referred in unquallified language to the strained relations which it asserted were existing between physicians and druggists; the salient cause being the habit of counter prescribing, coupled with the more vicious habit of substituting. Since then, if we may judge from the tone of the bulk of new literature being sent out, the substitution habit is shown to be the one great enemy overtopping all others, to successful medical practice.

We do not mean to assert that all pharmacists are given to the habit. On the contrary we believe a large majority of them to be entirely free from and above suspicion. Still the fact remains that substitution is practiced to such an extent as to engender anxiety and timidity on the part of prescribing physicians.

Persistent effort at substituting is but a commendation of the genuine product sought to be imitated, and the practicing physician is quick to recognize the fact. And, once recognizing it, his confidence in the genuine is strengthened, while at the same time he is forced into the unpleasant attitude of maintaining a constant weariness over his prescriptions.

As fairly typifying this condition we give below an extract from a letter from Dr. Bostick, of Galena, written Oct. 24th, 1893, to the Antikamnia Chemical Co. This letter is, by the way, a fair prototype. He says:

"I became dissatisfied some time since with the action, or rather non-action, of what I supposed to be Antikamnia. I began to look into the matter and discovered the druggist had been substituting in my prescriptions. I then had him get me tablets which I felt quite sure he, with any appliances he had, could not imitate; since which time I have been entirely satisfied with its action. I am satisfied that much *stuff* is sold and palmed off as Antikamnia, much to the detriment of your article, which has proven so very satisfactory to me. In many where quinine is indicated, I cannot prescribe it on account of its action on the brain, unless with Antikanmia, which seems to remove the objectionable feature."

The foregoing will surely justify all practitioners, where they may have cause to suspect they are being subjected to any such practices, in insisting upon the perfect integrity of *everything* they specify in their prescriptions. *The doctor has the highest and best right to insist that no worthless substitute be imposed upon his defenseless patient. He knows the specific effect of the genuine drug and knows equally well it cannot be successfully imitated.—Courier of Medicine*, Nov. 1893.

